# Anterior Vertebral Body Tethering for Skeletally Immature Patients with AIS: Indication for Spinal Fusion at Skeletal Maturity Is Not Obviated in 60% of Cases

**DOI:** 10.3390/jcm12123933

**Published:** 2023-06-08

**Authors:** Kiril V. Mladenov, Hans O. Pinnschmidt, Ralf Stücker

**Affiliations:** 1 Pediatric Orthopedic Department, Altona Children’s Hospital, Bleickenallee 38, D-22763 Hamburg, Germany; 2 Department of Orthopedics, University Medical Center Hamburg-Eppendorf, D-20246 Hamburg, Germany; 3 Center for Experimental Medicine, Institute for Medical Biometry & Epidemiology, University Medical Center Hamburg-Eppendorf, Chrisoph-Probst-Weg 1, D-20246 Hamburg, Germany

**Keywords:** adolescent idiopathic scoliosis, anterior vertebral body tethering

## Abstract

The role of anterior vertebral body tethering (aVBT) in obviating the need for spinal fusion in patients with AIS remains unclear, and a large amount of variation exists in the data among different studies. The present study aims to investigate and analyze what factors have a potential influence on aVBT outcome. Skeletally immature patients with AIS who underwent aVBT for scoliosis correction were followed up until skeletal maturity. The mean age at the time of surgery was 13.4 ± 1.1, and the mean follow-up time was 2.5 ± 0.5 years. The Cobb angle of the main curve was 46.6 ± 9° at the time of surgery and was significantly corrected to 17.7 ± 10.4° (*p* < 0.001) immediately postoperatively. A significant loss of correction was observed during the latest follow-up (Cobb angle 33.8 ± 18.7°; *p* < 0.001). An indication for spinal fusion at skeletal maturity was not obviated in 60% of the patients. The factors identified as having an influence on the outcome were preoperative bone age and the magnitude of the major curve. Patients with advanced bone age and larger curves were more likely to reach an indication for spinal fusion at skeletal maturity. In conclusion, no general recommendation for aVBT can be made for AIS patients. The method can be discussed as a treatment option in skeletally very immature preadolescent patients (Sanders Stadium ≤ 2) with a moderate Cobb angle (≤50°) who failed previous brace therapy.

## 1. Introduction

The purpose of treating adolescent idiopathic scoliosis (AIS) is to achieve a well-balanced, painless, functional spine with a curvature of less than 50° [1,2,3,4,5,6,7]. Based on observations of the natural history of untreated cases with continuous progression even after skeletal maturity [4,7,8], current treatment protocols recommend surgical treatment if the Cobb angle of the coronal curve is greater than 50°, with spinal fusion being the currently widely accepted method of choice for patients with advanced skeletal maturity or completed skeletal growth [9]. Good correction results, low complication rates, and high patient satisfaction have been reported following the use of this method in long-term studies [10,11]. However, the risk of functional impairment and symptomatic degeneration of adjacent segments due to decreased spinal mobility after spinal fusion are still major concerns, especially if lumbar segments are involved [11,12,13]. In recent years, anterior vertebral body tethering (aVBT) has been introduced as a fusionless surgical alternative for the treatment of patients with AIS [14,15], and clinical knowledge about aVBT has been continuously growing [16,17]. However, previously reported results have been very variable and even controversial between studies [18]. Moreover, clearly defined treatment protocols for aVBT are not yet available, and currently, no consensus exists between spinal surgeons concerning the indications and recommendations for aVBT [19].

The most important question about the role of aVBT in obviating the need for spinal fusion in AIS patients at maturity is currently unanswered, reflecting insufficient observations in patients who reached maturity after being previously treated with aVBT at a skeletally immature age. In addition, the importance of factors related to the curve characteristics and the skeletal maturity of the patient at the time of surgery or the impact of implant failure on the final outcome are still unclear.

The primary aim of this study was to evaluate if aVBT performed on immature patients with AIS can obviate an indication for spinal fusion at skeletal maturity. The secondary aims were to investigate what factors influence aVBT outcome and to study the indication for aVBT in patients with AIS.

## 2. Materials and Methods

This retrospective study on AIS patients treated by means of aVBT at a single institution was carried out between January 2018 and December 2022. The medical data for each subject was analyzed after it was collected from patient charts and serial X-rays. The Cobb angle measured on spinal radiographs was used to evaluate the curve magnitude. The curve type was defined according to the Lenke criteria and classification [20]. The Sanders Stadium (SS) [21] and the proximal femoral maturity index (PFMI) [22] were used to evaluate skeletal maturity. All relevant clinical data were reviewed by two orthopedic surgeons (K.M. and R.S.). The indications for aVBT at our institution were as follows:

- Patients with AIS who failed previous brace therapy;
- Preoperative Cobb angle of the major curve ≤ 65°;
- Preoperative Cobb angle of thoracic kyphosis ≤ 40°;
- Lenke curve types 1, 3, and 5;
- Skeletal immaturity at the time of surgery, with at least 2 years of residual growth corresponding to Sanders Stadium ≤ 5.

The inclusion criteria for participation in the study were follow-up until radiologically confirmed skeletal maturity corresponding to Sanders Stadium ≥ 7 or PFMI ≥ 6.

Patients who had previous spinal surgery at other institutions were excluded.

Serial spinal radiographs were measured preoperatively, immediately postoperatively, and at 6-month intervals until the latest follow-up. An indication for spinal fusion was defined as successfully prevented if the Cobb angle of the coronal curve measured ≤ 50° after skeletal maturity. If the Cobb angle was ≥51°, the outcome was defined as unsuccessful.

The following factors were analyzed for their possible influence on the final outcome after skeletal maturity was reached:

- Preoperative: bone age, Cobb angle of the major curve, instrumented segment of the spine. The patients were divided into subgroups according to bone age: Sanders Stadium (SS): ≤2 vs. ≥3; Cobb angle, major curve: ≤50° vs. ≥51°; instrumented segment of the spine: at or above T12 vs. at or below L1.
- Postoperative: Cobb angle, rupture of the flexible tether. Tether breakage was anticipated if, on serial X-rays, the divergence between the screws placed at two adjacent segments of ≥5° Cobb increased.

### 2.1. Statistical Analysis

Continuous and categorical data were descriptively presented as means and standard deviations and as category counts and category percentages, respectively. Paired t-tests were used to compare continuous variables at different time points, while χ^2^-tests, Fisher’s exact tests, odds ratios, and relative risk were used to examine the relationships between the categorical variables. All statistical analyses were performed using SPSS 29 (IBM^®^) software. The level of significance was set at *p* < 0.05. Since this is an explorative study, no adjustments were made for multiple tests.

### 2.2. Surgical Technique

aVBT was performed only for major curves, with the instrumentation extending from the upper to the lower end vertebra. For thoracic curves, aVBT was performed by means of an open convex-sided lateral thoracotomy through two separate 8–10-centimeter-long skin incisions with intermittent single-lung ventilation. For instrumentation extending to the lumbar spine, one thoracotomy and one open retroperitoneal approach through a separate 8–10-centimeter-long skin incision were performed. Bicortical vertebral body screws were placed in the convex to concave direction, and a flexible tether was attached to the heads of the screws beginning cranially. It was put under tension of 150 N between the two uppermost and the two lowermost instrumented segments and of 400 N between the periapical segments in order to achieve the best possible initial apical correction and to avoid decompensation of the adjacent non-instrumented segments. A chest tube was placed before soft tissue closure.

## 3. Results

### 3.1. Patient Demographics and Clinical and Radiological Characteristics

Since the introduction of aVBT at our institution in 2018, the procedure has been performed on a total of 32 skeletally immature patients. Ultimately, a total of 20 patients reached skeletal maturity during the follow-up period and were eligible for the present study. None of the patients were lost to follow-up. One patient received a primary aVBT procedure for both the thoracic and lumbar sections of their spine in two separate surgical procedures; thus, a total of twenty-one aVBT procedures were included in this study. The study group comprised five males and fifteen females. The mean age at the time of the index procedure was 13.4 ± 1.1 years (range: 10.5–15.3 years), and the mean follow-up time was 2.5 ± 0.5 years. There were six Lenke type 1 (30%), one Lenke type 3 (5%), and thirteen Lenke type 5 (65%) curves. The mean bone age at surgery corresponded to SS 3.2. There were five (25%) SS 1 patients; two (10%) SS 2 patients; seven (35%) SS 3 patients; and six (30%) SS 5 patients. All patients were skeletally mature at their latest follow-up, confirmed by bone age corresponding to Sanders Stadium ≥ 7 or PFMI ≥ 6. There were six aVBT instrumentations on only the thoracic spine and fourteen instrumentations on the lumbar spine (including one patient who received instrumentation on both the thoracic and lumbar sections of their spines). The cohort demographics are summarized in Table 1.

### 3.2. Radiological Results

The Cobb angle of the major curve measured 46.6 ± 9° preoperatively and was corrected to 17.7 ± 10.4° postoperatively, showing significant correction (*p* < 0.001). Upon further follow-up, a significant loss of the initially achieved curve correction was observed, with the Cobb angle measuring 33.8 ± 18.7° at the latest follow-up (*p* < 0.001).

The Cobb angle of the minor curve was 36.5 ± 12.3° at the time of surgery, 29.4 ± 14.4° immediately after aVBT, and 36.2 ± 23.5° at the latest follow-up, showing no statistically significant change (*p* > 0.05).

The mean thoracic kyphosis (TK) and lumbar lordosis (LL) measured 29.8 ± 7.9° and 50.5 ± 5.3° preoperatively and remained unchanged at the latest follow-up: TK, 29.5 ± 9.9°; LL, 48.7 ± 7.2° (*p* > 0.05).

Tether rupture according to the abovementioned criteria was anticipated in a total of 15 (75%) patients and occurred at an average of 20.8 months after aVBT surgery (range: 4–31 months). The indication for tether revision was critically evaluated for every individual based on expected remaining growth, and revision was performed only if bone age at that point corresponded to SS ≤ 5. Only one patient (5%) underwent revision surgery for early rupture of the tether, diagnosed 4 months after primary aVBT. At revision surgery, a “double tether aVBT” was performed. Twelve months after revision surgery, the patient complained of sudden low back pain, and the X-ray showed a fracture in the lowermost vertebral screw placed during revision surgery. Since pain completely subsided without treatment and bone maturity was advanced at this point (SS 7), no further tether revision was indicated.

Overcorrection was observed in only one patient who was very skeletally immature (SS1) at the time of aVBT surgery. The tether was surgically released 20 months after aVBT, and the Cobb angle remained stable during further follow-up.

The coronal and sagittal Cobb angle values are summarized in Table 2.

After skeletal maturity, the Cobb angle was found to be ≥51° in 12 patients, indicating that aVBT was “unsuccessful” at preventing an indication for spinal fusion in 60% of the cases. At the time of data collection, spinal fusion had already been performed in four patients and was still pending in eight others. In five of the twelve patients with an indication for spinal fusion, significant worsening of the non-instrumented minor curve with a Cobb angle ≥ 51° was observed.

### 3.3. Evaluation of Risk Factors with Influence on Outcome

#### 3.3.1. Bone Age at Surgery

A bone age corresponding to SS ≤ 2 was set as the “cut-off” criterion for substratification because this bone age defines the period before the onset of rapid growth. When substratified for bone age at surgery, patients with SS ≤ 2 (n = 7 (35%)) compared with those with SS ≥ 3 (n = 13 (65%)) showed similar preoperative (41.3 ± 9.4° vs. 49.3 ± 7.3°; *p* > 0.05) and immediately postoperative (14.2 ± 6.5° vs. 19.5 ± 11.7°; *p* > 0.05) Cobb angles. Upon further follow-up, the initially achieved Cobb angle correction in the very skeletally immature group (SS ≤ 2) remained stable, with a mean Cobb angle measuring 13.5 ± 13.8° at the latest follow-up, whereas in the less skeletally immature patients (SS ≥ 3), a significant loss of correction, with a Cobb angle at the latest follow-up measuring 43.8 ± 10.8° (*p* < 0.001), was observed. Table 3 presents the longitudinal Cobb angle values in the SS ≤ 2 and SS ≥ 3 subgroups.

The indication for spinal fusion at skeletal maturity in the SS ≤ 2 subgroup was considerably lower, at 42% (3/7), compared with that in the SS ≥ 3 subgroup, at 69% (9/13); however, the difference did not reach statistical significance.

#### 3.3.2. Preoperative Cobb Angle of the Major Curve 

When substratified for the size of the major curve before surgery, the preoperative Cobb angle measured ≤ 50° in twelve patients (60%) and ≥51° in eight patients (40%). At skeletal maturity, spinal fusion was indicated in 41% (5/12) of the patients with an initial Cobb angle ≤ 50° and in 87% (7/8) of those with an initial Cobb angle ≥ 51°. Patients with a preoperative Cobb angle ≤ 50° were found to have a lower risk of reaching an indication for fusion at maturity (odds ratio 0.44; 95% confidence interval 0.211–0.917). In the subgroup with an initial Cobb angle ≥ 51°, the relative risk of reaching an indication for spinal fusion at maturity was 47% higher compared with that in patients with smaller initial curves.

#### 3.3.3. Location of the Instrumented Curve

When substratified for location of the instrumented curve, spinal fusion at skeletal maturity was indicated in 42% (3/7) of the cases with instrumentation of the thoracic spine and in 64% (9/14) of those who underwent instrumentation of the lumbar spine. The relative risk of reaching an indication for spinal fusion at skeletal maturity was not significantly different between the two subgroups (*p* = 0.319).

#### 3.3.4. Tether Rupture

When substratified for tether rupture, spinal fusion at maturity was indicated in 75% (9/12) of patients with a radiologically detected ruptured tether. In the five patients without tether rupture, spinal fusion was indicated in three (60%). The relative risk of reaching an indication for spinal fusion at skeletal maturity was not statistically different between the two subgroups (*p* = 0.154).

## 4. Discussion

In the present study, we found that in 60% (12/20) of the skeletally immature AIS patients who underwent aVBT surgery, the indication for spinal fusion at skeletal maturity was not obviated. The need for spinal fusion after aVBT was already reported in previous studies at a variable rate, being 0% at 1 year and becoming increasingly higher with the duration of postoperative observation, reaching 40% at 4 years of follow-up [16]. Spinal fusion was already performed in four patients and indicated but still pending in another eight patients in our series, indicating that aVBT was unsuccessful at preventing the indication for spinal fusion at skeletal maturity in more than half of our cohort when only the major curve was addressed with aVBT. These findings contrast the results of the study by Horschemeyer et al. [23], in which fusion was prevented in 93% of the patients. This discrepancy could be an artifact of the smaller initial curve and instrumentation of mostly thoracic curves in their study or other confounding factors such as revision of the tether (reported as 21%), which may have postponed loss of the correction due to a prolonged internal “bracing” effect of the tether. 

We chose a coronal curve ≥ 51° as the cut-off for “unsuccessful” outcomes since this corresponds to the currently widely accepted indication criteria for spinal fusion after full skeletal maturity development [9]. Of note is that the initially achieved significant correction of the major curve was substantially lost with further follow-up, reaching an indication for spinal fusion in 60% of our cohort. Nineteen patients in our series had aVBT for a single curve, and only one patient presenting initially with a Lenke type 3 curve had aVBT for both major curves. The curve type evaluation (major/minor) in our study was performed according to the Lenke criteria, and tethering was performed only on the “major” curves in our cohort. However, in five of the twelve patients with an indication for spinal fusion at maturity in our study, significant worsening of the minor curve was observed during follow-up. The deterioration of the “minor” curve in those cases was observed at an advanced stage of skeletal maturity, which precluded the possibility of performing aVBT. A “prophylactic” approach in patients with aVBT performed on the minor curve would lead to overtreatment in many cases and was not elected. 

The rationale behind aVBT for the treatment of AIS and the anticipated advantage of this motion-preserving method over spinal fusion would be expected only if sustainable scoliosis correction is achieved and maintained in the long run, thus the need for spinal fusion. An initial curve correction with aVBT is achieved through the “bracing effect” of the convex flexible tether, but long-term maintenance of the correction or further improvement in the Cobb angle can be expected only if a substantial alteration in the vertebral morphology and/or the intervertebral disc occurs by means of growth modulation. Otherwise, curve correction will be lost once the “bracing effect” of the tether subsides. However, the flexible tether, as a non-biological structure without regenerative potential, will rupture at some point under the influence of wear and tear. Therefore, growth modulation is of critical importance for long-term outcomes. Growth modulation of the spine has been observed in experimental studies on healthy animals [24]. However, there is a paucity of published clinical data on vertebral growth modulation after aVBT in immature AIS patients, with the data being inconclusive and very controversial throughout the studies [18,25]. The curve behavior after aVBT during follow-up has not been uniform between patients or between studies. Different patterns were described, varying from further significant curve improvement to significant curve deterioration [23,26]. Obviously, this is due to the inconsistent and unpredictable growth-modulating effect of the tethering that Newton et al. [27] reported on a peri-apical growth-modulating effect after aVBT in immature AIS patients with Sanders Stadium 2–3. In a study comparing seven patients in whom growth modulation occurred—defined as “responders”—and seven other patients with identical bone age in whom no growth modulation occurred—defined as “non-responders”, concave vertebral growth at 0.13 mm/segment/month occurred in the “responders” group compared with that at 0.05 mm/segment/month in the “non-responders” group. The observed disc wedging and coronal angulation in the “responders” measured 0.11 and 0.12 mm, respectively, and were significantly higher than those of the “non-responders”, in which much lower growth, 0.02 and 0.04 mm, respectively, was observed. Notably, the information from this study is of very limited value since the factors potentially influencing being a “responder” or “nonresponder” were not reported.

Mc Donald et al. [28] studied the growth modulation effect after thoracic aVBT in 51 immature AIS patients with Sanders Stadium 2–4. Measurements were performed on 764 vertebrae and 807 discs. The authors concluded that “when correction occurred”, this growth modulation happened through a reduction in the convex disc height and via differential vertebral growth, with concave growth being 1.3 times greater than convex growth. However, the clinical value of these observations is very limited since patients with skeletal maturity corresponding to Sanders Stadium > 4, as well as those in which growth modulation failed and those with ruptured tethers, were excluded from the study. Based on these data, no conclusion can be drawn about the factors that influence growth modulation.

Our observations confirm that the amount of residual growth plays an important role in the final outcome, since the patients in our cohort in whom spinal fusion was not indicated at skeletal maturity were very skeletally immature at the time of aVBT surgery. However, in contrast to Alanay et al. [29], who reported on the maintenance of or further improvement in Cobb angle of the major curve after aVBT in patients with Sanders Stadium ≤ 3, we observed that the initially achieved curve correction after aVBT remained stable or improved slightly upon further follow-up only in very skeletally immature patients who are in the “preadolescent slow” maturity stadium, corresponding to Sanders Stadium ≤ 2. On the contrary, in patients with Sanders Stadium ≥ 3 (“adolescent rapid early”), no further correction but instead continuous, significant loss of correction was observed during follow-up. 

The indication for spinal fusion at skeletal maturity in our study was considerably higher (69%) when aVBT was performed after the rapid pubertal growth phase was reached (corresponding to SS ≥ 3), compared with 42% before the onset of rapid skeletal growth (SS ≤ 2). However, a comparison between the subgroups failed to show a significantly lower risk for “unsuccessful” outcomes when aVBT was performed in very immature patients before the onset of rapid growth. 

In a multicenter study, aVBT was performed in patients with Cobb angles up to 85° [18]. Newton et al. [30] recommended aVBT for patients with Cobb angles of ≤65° and stated that fusion is the preferred approach for larger curves in order to achieve sustainable correction. We chose a Cobb angle of 50° as the “cut-off” criteria for substratification for coronal curve magnitude since the decision to proceed with surgery is commonly based on this value. Our results identified that the Cobb angle of the major curve is a factor that influences the results of aVBT since 87% of the patients with initial curves of ≥51° had “unsuccessful” outcomes at skeletal maturity compared with only 42% of those with a Cobb angle of ≤50°. Accordingly, patients with greater preoperative Cobb angles had a 47% higher risk of being a candidate for spinal fusion at maturity.

The indication for aVBT depending on the location of the apex of scoliosis is controversially discussed, with some authors recommending aVBT preferably for single thoracic curves [30] and others performing aVBT for lumbar curves as well [31]. Newton et al. reported significantly better curve corrections and more sustainable results with posterior spinal fusion compared with aVBT for thoracic curves in AIS patients [32]. Furthermore, the preservation of lumbar spine mobility would obviously provide a greater functional benefit to the patient compared with thoracic motion preservation. Thus, despite previous findings identifying lumbar curves as one of the risk factors for tether breakage [33], our indication for aVBT was preferably for the lumbar spine, and most of the patients in our study underwent aVBT for lumbar curves. In our study, there was a tendency for a higher rate of “unsuccessful” outcomes when aVBT was performed for lumbar curves compared with when aVBT was performed for thoracic curves (64% vs. 42%); however, the difference did not reach statistical significance. 

Tether rupture after aVBT has been observed at a variable rate in up to 48% of patients [23]. Accordingly, the revision rate for tether replacement has been increasingly reported as being concordant with the duration of follow-up [33,34]. We used the >5° rule to define a tether rupture [35], since the false-positive rate with this method was reported to be very low [35]. Even if it was previously reported that only 56% of the breakages could be seen on native radiographs, a “missed” tether breakage without loss of correction is of limited clinical importance since this does not result in an indication for revision [35]. We observed a rupture in the tether in 15 of 20 (75%) of our patients, which was diagnosed at a mean of 20.6 months after aVBT surgery. Since ruptures can be asymptomatic, they can only be detected by imaging performed during a scheduled follow-up visit. Thus, the time of rupture can only be estimated. We did not routinely perform revision surgery for tether ruptures; instead, decisions were made on an individual basis depending on the residual skeletal growth of the patient. In patients with advanced skeletal maturity (SS ≥ 5), a tether revision was not performed since no relevant growth-modulating effect of the tether could be expected. Only 1 out of 20 (5%) patients in our study had revision surgery for early rupture of the tether, diagnosed 4 months after primary aVBT.

As reported by Baronchini et al., greater curve magnitude and lumbar apex were two of the risk factors related to tether rupture [33]. Baronchini et al. proposed a double band technique in order to decrease the risk of tether breakage when addressing a lumbar curve with aVBT. Interestingly, the preliminary results failed to demonstrate the long-term benefits of this approach [31]. Based on our observations on tether breakage occurring at a mean of 20 months after primary aVBT with a single tether and the lack of impact on final outcome, we cannot recommend a double band technique at initial surgery since tether ruptures occur mostly after the time for the growth-modulating effect has elapsed and tether ruptures due to mechanical wear inevitably occur at some point, even with a double tether. Additionally, the potential negative effects on the intervertebral disk due to the higher “rigidity” of the instrumentation pose a concern. In our study, tether rupture was not a significant risk factor for an “unsuccessful” result since the number of patients with and without tether rupture who reached an indication for spinal fusion at skeletal maturity was similar (75% vs. 60%). 

Growing evidence suggests that a prediction of curve progression in AIS depends not only on clinical and radiological factors, and the importance of genetic and epigenetic factors that influence prognosis has recently been identified [36]. Although promising, current data are still insufficient to guide clinical decisions, and this field should be the object of future investigation.

Our study limitations include the retrospective design and the small number of patients, which can be a source of statistical “underpower”. However, the strengths of our study are the uniform indication criteria used for aVBT, the standardized surgical technique, the availability of complete clinical and radiographic data for all the patients until radiologically documented skeletal maturity, and the lack of patients lost to follow-up. 

## 5. Conclusions

No general recommendations for aVBT for the treatment of skeletally immature patients with AIS can currently be given since outcomes are unpredictable and more than half of the patients will be candidates for spinal fusion after skeletal maturity is reached.

Skeletal maturity and the Cobb angle of the major curve at the time of aVBT surgery are factors that influence the final result, with advanced skeletal maturity and a larger Cobb angle having negative impacts on the final outcome.

Anterior vertebral body tethering can be discussed as a treatment option in very skeletally immature preadolescent AIS patients (SS ≤ 2) with moderate deformity and Cobb angle (≤50°) who failed previous brace therapy.

## Figures and Tables

**Table 1:** Cohort demographics (n = 20).

Cohort Demographics (n = 20)				
Gender	Male n = 5 (25%)			Female n = 15 (75%)
Age	At surgery 13.4 ± 1.1			At the latest follow-up 15.9 ± 1
Lenke curve type	1	3		5
Nr. Patients	6 (30%)	1 (5%)		13 (65%)
Sanders Stadium at the time of surgery	1	2	3	5
No. of patients	2 (10%)	5 (25%)	7 (35%)	6 (30%)

**Table 2:** Data for coronal and sagittal Cobb angles.

	Cobb°			*p* Value		
	Preoperative	First Erect	Last f/u	Preop. vs. First Erect	First Erect vs. Last f/u	Preop. vs. Last f/u
Major curve (coronal)	46.6 ± 9°	17.7 ± 10.4°	33.8 ± 18.7°	<0.001	<0.001	>0.05
Minor curve (coronal)	36.5 ± 12.3°	29.4 ± 14.4°	36.2 ± 23.5°	>0.05	>0.05	>0.05
Thoracic kyphosis	29.8 ± 7.9°		29.5 ± 9.9°			>0.05
Lumbar lordosis	50.5 ± 5.3°		48.7 ± 7.2°			>0.05

**Table 3:** Cobb angle of major curves with longitudinal data after aVBT according to bone age at surgery.

Major Curve Cobb°	Preoperative	First Erect Postoperative	Follow-Up (Mo)				*p*-Value First Erect Postoperative vs. Last f/u
			6	12	18	Last f/u	
Sanders ≤ 2	41.3 ± 9.4°	14.2 ± 6.5	15.5 ± 11.3	12.1 ± 17.3	18.6 ± 9.7	13.5 ± 13.8	*p* = 0.474
Sanders ≥ 3	49.3 ± 7.3°	19.5 ± 11.7	23.8 ± 12.2	30.2 ± 11.1	36.6 ± 12.0	43.8 ± 10.8	*p* < 0.001

## Data Availability

All medical data are available on request and will be provided if needed by the corresponding author.
